# Supplementary material for: The causal role of circulating inflammatory markers in osteoporosis: a bidirectional Mendelian randomized study
Source: Front Immunol. 2024 Jul 18;15:1412298. doi: 10.3389/fimmu.2024.1412298 (PMC11291241; doi:10.3389/fimmu.2024.1412298)
Supplement: Supplementary file 1 [file DataSheet_1.zip › supplementary material/OSTEOPOROSIS-CIM/GCST90274823.tsv.gz_forest.pdf]

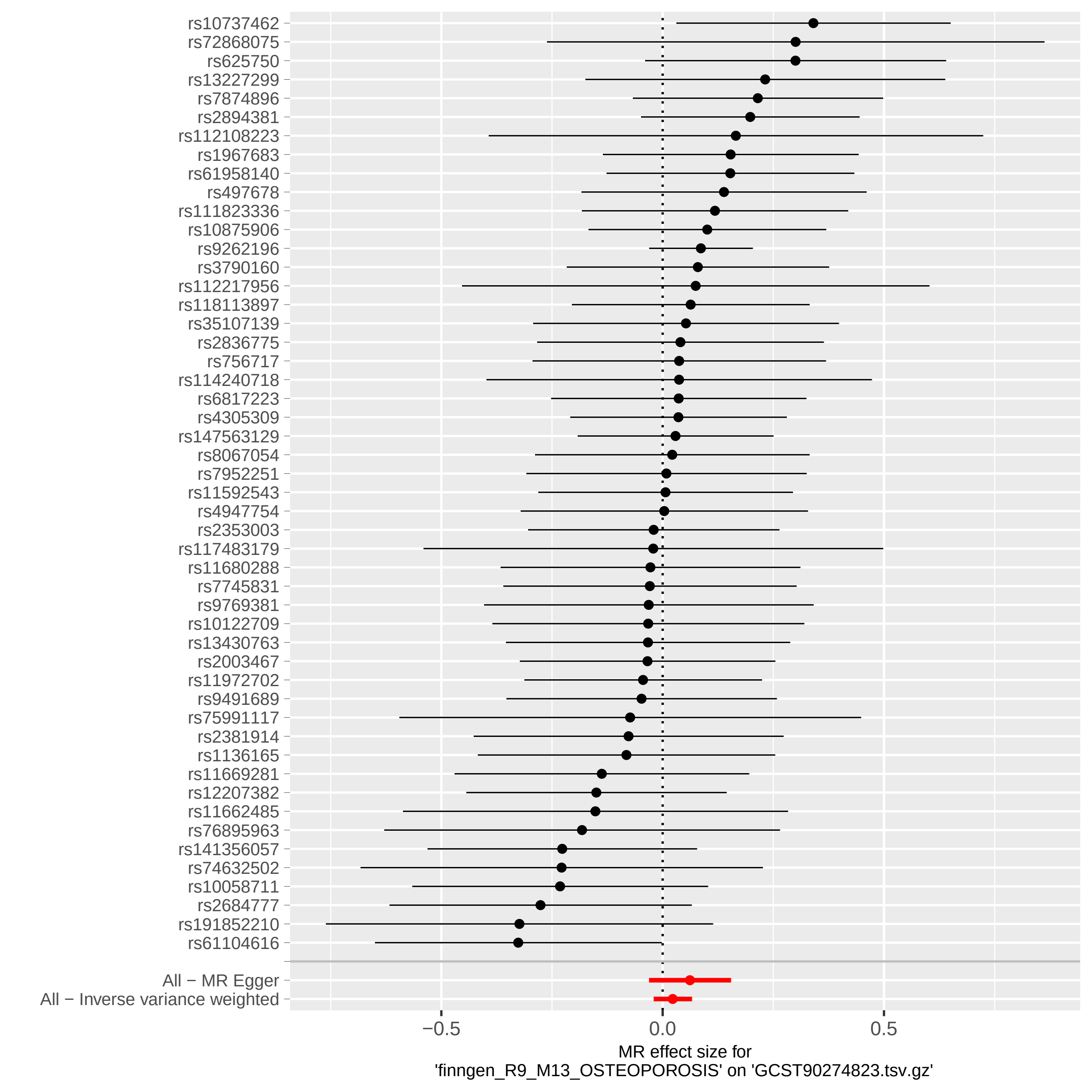

rs10737462
rs72868075
rs625750
rs13227299
rs7874896
rs2894381
rs112108223
rs1967683
rs61958140
rs497678
rs111823336
rs10875906
rs9262196
rs3790160
rs112217956
rs118113897
rs35107139
rs2836775
rs756717
rs114240718
rs6817223
rs4305309
rs147563129
rs8067054
rs7952251
rs11592543
rs4947754
rs2353003
rs117483179
rs11680288
rs7745831
rs9769381
rs10122709
rs13430763
rs2003467
rs11972702
rs9491689
rs75991117
rs2381914
rs1136165
rs11669281
rs12207382
rs11662485
rs76895963
rs141356057
rs74632502
rs10058711
rs2684777
rs191852210
rs61104616
All − MR Egger
All − Inverse variance weighted
−0.5
0.0
0.5
MR effect size for
'finngen_R9_M13_OSTEOPOROSIS' on 'GCST90274823.tsv.gz'
